# Does persistence to methotrexate treatment in early rheumatoid arthritis have a familial component?

**DOI:** 10.1186/s13075-022-02873-z

**Published:** 2022-08-06

**Authors:** Anton Öberg Sysojev, Thomas Frisell, Bénédicte Delcoigne, Saedis Saevarsdottir, Johan Askling, Helga Westerlind

**Affiliations:** 1 Clinical Epidemiology Division, Department of Medicine Solna, Karolinska Institute, Stockholm, Sweden; 2 Faculty of Medicine, School of Health Sciences, University of Iceland, Reykjavik, Iceland; 3 Rheumatology, Theme Inflammation & Ageing, Karolinska University Hospital, Stockholm, Sweden

**Keywords:** Rheumatoid arthritis, Methotrexate, Heritability, Familiality, Treatment persistence

## Abstract

**Objectives:**

To assess whether persistence to treatment with methotrexate (MTX) in early rheumatoid arthritis (RA) is shared among first-degree relatives with RA and to estimate any underlying heritability.

**Methods:**

First-degree relative pairs diagnosed with RA 1999–2018 and starting MTX (in monotherapy) as their first disease-modifying anti-rheumatic drug (DMARD) treatment were identified by linking the Swedish Rheumatology Quality Register to national registers. Short- and long-term persistence to MTX was defined as remaining on treatment at 1 and 3 years, respectively, with no additional DMARDs added. We assessed familial aggregation through relative risks (RR) using log-binomial regression with robust standard errors and estimated heritability using tetrachoric correlations. We also explored the familial aggregation of EULAR treatment response after 3 and 6 months. To mimic the clinical setting, we also tested the association between having a family history of MTX persistence and persistence within the index patient.

**Results:**

Familial persistence was not associated with persistence at 1 (RR=1.02, 95% CI 0.87–1.20), only at 3 (RR=1.41, 95% CI 1.14–1.74) years. Heritability at 1 and 3 years was estimated to be 0.08 (95% CI 0–0.43) and 0.58 (95% CI 0.27–0.89), respectively. No significant associations were found between family history and EULAR response at 3 and 6 months, neither overall nor in the clinical setting analysis.

**Conclusions:**

Our findings imply a familial component, including a possible genetic element, within the long-term persistence to MTX following RA diagnosis. Whether this component is reflective of characteristics of the underlying RA disease or determinants for sustained response to MTX in itself will require further investigation.

## Introduction

In rheumatoid arthritis (RA), early and effective treatment reduces inflammation, prevents joint damage, and improves quality of life [1]. For the typical newly diagnosed patient, most treatment guidelines currently suggest methotrexate (MTX) in DMARD monotherapy as first-line treatment [2, 3]. However, this approach does not take the heterogeneity of RA and treatment responsiveness into account; at 3 months, only ~30% of patients initiating MTX in DMARD monotherapy have achieved a good response [4, 5], and only two-thirds of patients remain on this regimen at 1 year [6]. As treatment guidelines suggest a treat-to-target approach, those unable to reach the target require treatment with other DMARDs, or combination therapy [2, 3]. Thus, early-stage identification of patients with a low chance of *remaining on initial treatment* (here: MTX in DMARD monotherapy) is of utmost importance as these individuals should be offered alternative therapies up-front.

Previous studies have identified several factors, genetic as well as clinical, that associate with *primary response* to MTX, although they have generally either been weak predictors or predictors that call for replication [5, 7–10]. Most of these studies have used disease activity scores and their components, which often suffer from missing data, as treatment outcome. Few studies have studied *treatment persistence* as the outcome, which, in a treat-to-target paradigm should serve as a proxy for favorable tolerance and sustained treatment response. Using persistence as the outcome measure, a previous study from our group found that family history of RA, *in itself*, was unable to predict persistence to MTX in DMARD monotherapy at 3 and 6 months, though that study did not take family history of MTX treatment response into account [11].

Genetic factors contributing to MTX treatment response have been extensively studied with multiple genetic variants found to be associated with MTX efficacy [8, 9]. Studies have further indicated that models including genetic factors predict MTX treatment response better than those relying solely on clinical factors [12, 13]. This would imply the existence of a non-negligible genetic component and, per extension, a familial component to MTX treatment response. Whereas the existence of a familial component in the etiology of RA has been well documented [14–16], evidence of a familial component within aspects of the clinical presentation of the disease, such as disease severity and treatment response, has so far been limited [11, 17, 18].

Evidence of a familial aggregation would potentially allow for early identification of patients who are less likely to respond to and remain well on MTX through assessment of their family history of this response. Furthermore, in clinical practice, questions on the information contained within a family history of a certain treatment outcome are not infrequent, yet the evidence to back up the response to such questions is scarce.

In this study, we aimed to assess whether a familial component contributes to the persistence to MTX used as DMARD monotherapy in early RA. We aimed to do this by testing whether treatment persistence aggregates within families of first-degree relatives concordant for RA and treatment with MTX and to quantify its magnitude by estimating the corresponding heritability.

## Methods and materials

### Materials

We used data from the Swedish Rheumatology Quality Register (SRQ) linked to other national registries by the unique personal identity number issued to all permanent residents of Sweden [19]. Established in 1996, SRQ is a nationwide clinical quality register of patients with inflammatory arthritis including individuals aged 18 and above. The register has above 85% coverage for all prevalent patients with RA in Sweden [20]. SRQ contains extensive baseline and longitudinal information including clinical data as well as information on prescribed drug treatments. We linked SRQ to the Multi-Generation Register (MGR), a Swedish national register containing information on parenthood for residents born after 1931 and living in Sweden since 1961, allowing for identification of pairs of first-degree relatives with RA. The coverage of MGR is high, with near-perfect coverage for individuals born in Sweden after 1961 [21]. We further linked data to the National Patient Register (NPR) and the Prescribed Drug Register (PDR) for validation of patient inclusion/exclusion criteria for sensitivity analysis. NPR is a nationwide register containing information on inpatient treatments since 1964 and outpatient visits to specialist care since 2001 [22]; PDR is a nationwide register containing information on dispensed drug prescriptions since July 2005 [23].

**Fig. 1:**
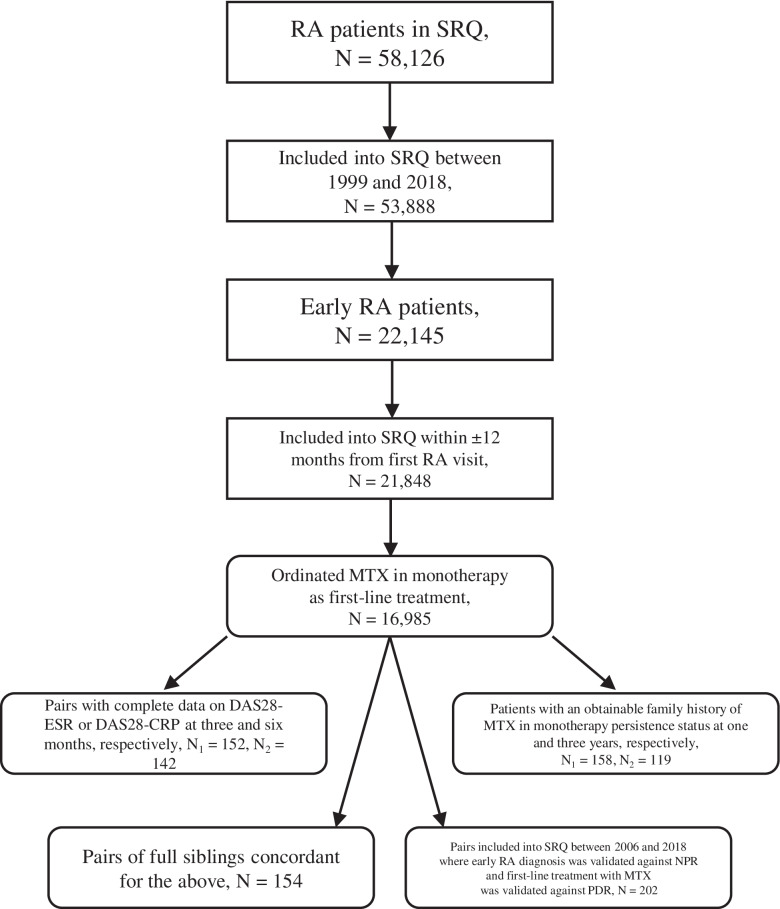
Visualization of inclusion/exclusion criteria employed to obtain the main cohort, the two exploratory analysis cohorts as well as the two sensitivity analysis cohorts. CRP, C-reactive protein; DAS, disease activity score; ESR, erythrocyte sedimentation rate; MTX, methotrexate; NPR, National Patient Register; PDR, Prescribed Drug Register; RA, rheumatoid arthritis; SRQ, Swedish Rheumatology Quality Register

Our cohort consisted of first-degree relative pairs concordant for early RA with MTX in DMARD monotherapy as their first prescribed treatment. Study inclusion was restricted to individuals born after 1931 and included in SRQ between 1999 and 2019. Early RA was defined as having symptom onset less than 12 months prior to diagnosis, where the diagnosis was ascertained by the treating rheumatologist per the ACR1987 or EULAR2010 criteria [24, 25]. We excluded all individuals who, in NPR, had their first ever visit listing RA more than 365 days before their SRQ inclusion date. Furthermore, patients were only included if they had been treated with MTX as their first ever DMARD, in monotherapy (defined as with no other DMARD prescriptions within 30 days of the first MTX prescription yet allowing MTX in combination with oral or intra-articular glucocorticoids, and nonsteroidal anti-inflammatory drugs). Lastly, we excluded all patients who did not have a first-degree relative fulfilling all of the above criteria (Fig. 1).

Persistence to MTX in DMARD monotherapy was defined as a binary variable at two follow-up time-points: persistence at 1 and 3 years, in analogy with a previous study of ours on persistence to MTX [6]. Individuals were considered persistent if they were still treated with MTX in DMARD monotherapy at 1 and 3 years, respectively, after the start of their first prescription in SRQ, with no other DMARD prescriptions allowed during this time period.

### Statistical analysis

Persistence to MTX in DMARD monotherapy in the first-degree relative was treated as exposure with persistence status at the time of interest (i.e., at 1 and 3 years) as outcome. Familial risks of treatment persistence were estimated using a log-binomial regression model [26]. Covariates adjusted for included sex, age, and year of diagnosis. A robust sandwich variance estimator was used to account for familial clustering whenever data included patients with more than one first-degree relative. Narrow-sense heritability for persistence to treatment with MTX in DMARD monotherapy was estimated by doubling the observed tetrachoric correlations [27, 28]. Out-of-bounds confidence intervals were truncated to 0/1 and are here reported with an asterisk.

The study cohort was extracted from a larger register linkage, using SAS (v9.4). Statistical analyses were performed in R (v4.0.2) [29]. Tetrachoric correlations were computed using the “polycor” package (v0.7-10) [30]; log-binomial regression models were fitted using the “logbin” package (v2.0.4) [31], and robust standard errors were computed using the “sandwich” package (v3.0.0) [32]. This study was approved by the Stockholm ethical review board (DNR 2015/1844-31).

### Sensitivity analysis

In addition to the above-described analyses, we extracted two secondary cohorts for sensitivity analyses to assess the robustness of our main findings. In the first sensitivity analysis, we restricted our cohort of first-degree relative pairs to pairs consisting exclusively of full siblings as these are expected to share more environment. In the second sensitivity analysis, we kept only individuals included in SRQ between 2006 and 2018, where both the early RA diagnosis and the first-line treatment with MTX in DMARD monotherapy could be fully validated against NPR and PDR [22, 23]. As persistence may be a heterogeneous treatment outcome during the study period used in the main analysis, the latter sensitivity analysis should improve validity through increased homogeneity of cohort participants. Here, this meant excluding individuals who had their first visit listing RA as the main diagnosis in NPR more than 12 months before their SRQ inclusion date, as well as individuals, either prescribed MTX > 90 days before their first SRQ prescription of MTX or prescribed a non-MTX DMARD in PDR. Additionally, persistence status was further validated by classifying patients as non-responders if they had received any prescription of a non-MTX DMARD, per PDR, during the study period. The statistical analyses described above were repeated in both sensitivity cohorts.

### Exploratory analysis

We performed two exploratory analyses to investigate familial aggregation of MTX treatment *response*. Firstly, to further assess short-term associations, we investigated the familial aggregation of EULAR DAS28 response at 3 and 6 months, using data from SRQ on DAS28-ESR and DAS28-CRP (if response status by DAS28-ESR was missing) [33–35]. Here, baseline DAS28 (recorded at the visit closest to the date of treatment start from a period of 90 days before to 30 days after start of treatment) was compared with DAS28 at 3 months (recorded at the visit closest to 90 days after treatment start during days 31–149) and 6 months (recorded at the visit closest to 180 days after treatment start during days 150–269). Patients were considered responders if they achieved a good or moderate EULAR (DAS28-ESR or DAS28-CRP) response at the time of evaluation. Patients achieving neither good nor moderate EULAR response, or who stopped treatment with MTX in DMARD monotherapy (either due to discontinuation of MTX or prescription of additional DMARDs), were considered non-responders.

Secondly, we explored the predictive capabilities of persistence to treatment with MTX in DMARD monotherapy, as a family history variable, this being what would be measurable in a clinical setting. Here, a positive family history meant that, at the time of the patient’s treatment start, they had a first-degree relative who had started treatment 1 (or 3, respectively) years earlier and were still persistent on that treatment. Correspondingly, having a negative family history meant that the corresponding first-degree relative that started treatment 1 or 3 years prior was not persistent. As a result of this, all patients starting treatment with MTX in DMARD monotherapy less than 1 (or 3, respectively) years before their first-degree relative did so were ineligible for this analysis.

Due to parameter estimation convergence issues with the log-binomial regression, logistic regression was employed to fit the exploratory analysis data. Covariates adjusted for included sex, age, and year of diagnosis where age was categorized as <50, ≥50 and <65, and ≥65 years at treatment start. Otherwise, statistical analyses of the exploratory sub-cohorts were identical to what was described in the above section.

## Results

We identified 347 unique individuals with at least one first-degree relative concordant for early RA and treatment with MTX in DMARD monotherapy. Together, these 347 individuals constituted 354 distinct pairs of first-degree relatives, making up the study cohort for the main analysis (Fig. 1). The majority of relative pairs were siblings (44%), followed by equal shares of offspring (28%) and parents (18% mothers and 10% fathers). Mean age at treatment start was 58 years (IQR: 47–69), and 246 (71%) of all individuals were female.

Among the 347 unique individuals, 231 (67%) were still on MTX in DMARD monotherapy at 1 year, and 175 (50%) at 3 years. Earlier age at treatment start and a greater fraction of female patients were observed among the non-persistent individuals, but the groups had similar proportions with seropositive RA and a similar number of identified first-degree relatives (Table 1). Among the 354 pairs of first-degree relatives, in 158 (45%), both individuals were persistent at 1 year, with 106 (30%) pairs wherein both individuals were persistent at 3 years.

**Table 1:** Demographics of the unique individuals within the study cohort consisting of Swedish early RA patients diagnosed 1999–2019, treated with MTX in monotherapy as their first prescribed DMARD and with a first-degree relative concordant for early RA and treatment with MTX in DMARD monotherapy; stratification by treatment persistence status

	MTX persistence at 1 year		MTX persistence at 3 years	
	Persistent, *N* = 231	Non-persistent, *N* = 116	Persistent, *N* = 175	Non-persistent, *N* = 172
Female (%)	159 (69%)	87 (75%)	117 (67%)	129 (75%)
Seropositive (%)	169 (75%)^a^	86 (75%)^b^	126 (74%)^a^	129 (75%)^b^
Glucocorticoids at baseline (%)	112 (48%)	51 (44%)	80 (46%)	83 (48%)
Mean age at the start of MTX (SD)	60 (14)	53 (15)	62 (13)	53 (15)
Median year of the start of MTX (Q1–Q3)	2012 (07–16)	2011 (06–15)	2013 (08–16)	2011 (07–15)
Median overall number of FDRs identified (Q1–Q3)	4 (3–5)	4 (3–5)	4 (3–5)	4 (3–5)
RR (95% CI)	1.02 (0.87–1.20)		1.41 (1.14–1.74)	
*h*^2^ (95% CI)	0.08 (0*–0.43)		0.58 (0.27–0.89)	

*DMARD* disease-modifying anti-rheumatic drug, *FDR* first-degree relative, *MTX* methotrexate, *RA* rheumatoid arthritis

*Truncated confidence interval boundary

^a^Five of these had missing status

^b^One of these had missing status

Familial persistence was not associated with persistence at 1 year (RR=1.02, 95% CI 0.87–1.20) but a significant association with persistence was observed at 3 years (RR=1.41, 95% CI 1.14–1.74). Narrow-sense heritability was estimated to be 0.08 (95% CI 0*–0.43) for persistence at 1 year, and 0.58 (95% CI 0.27–0.89) at 3 years.

### Exploratory analysis

Among the 354 first-degree relative pairs in our study cohort, 152 (43%) pairs had data on DAS28-ESR or DAS28-CRP at both baseline and 3 months, and 142 (40%) of the pairs had complete data at both baseline and 6 months. Among the former, 78 (51%) of pairs were concordant for being responders (i.e., both individuals achieved a good or moderate EULAR response and remained on treatment) while for EULAR response at 6 months, 42 (30%) of pairs were concordant for being responders. Distributions of patient characteristics were similar to those observed within the main cohort with more female patients and an earlier average age at disease onset among non-responders (Table 2). We found no evidence of a familial aggregation within having a good or moderate EULAR primary response to treatment, neither at 1 (OR=0.60, 95% CI 0.23–1.58) nor at 6 months (OR=0.71, 95% CI 0.36–1.41).

**Table 2:** Demographics of the unique individuals and ORs for the familial risk of having a EULAR response at 3 and 6 months, as well as being persistent at 1 and 3 years given a family history of persistence, both in a cohort of Swedish early RA patients diagnosed 1999–2019, treated with MTX in monotherapy as their first prescribed DMARD and with a first-degree relative concordant for early RA and treatment with MTX in DMARD monotherapy; stratified by response and persistence status respectively, for those included in the analysis

	EULAR response				Family history			
	EULAR response at 3 months		EULAR response at 6 months		Persistence at 1 year		Persistence at 3 years	
	Good or moderate responders, *N* = 156	Non-responders, *N* = 62	Good or moderate responders, *N* = 116	Non-responders, *N* = 92	Persistent, *N* = 108	Non-persistent, *N* = 50	Persistent, *N* = 56	Non-persistent, *N* = 63
Female (%)	107 (69%)	49 (79%)	77 (66%)	71 (77%)	74 (69%)	36 (72%)	36 (64%)	47 (75%)
Seropositive (%)	117 (76%)^a^	50 (81%)	87 (77%)^a^	67 (74%)^c^	73 (70%)^a^	34 (69%)^c^	38 (70%)^b^	43 (69%)^c^
Glucocorticoids at baseline (%)	74 (48%)	31 (47%)	65 (57%)	38 (40%)	54 (50%)	17 (34%)	23 (41%)	31 (49%)
Mean age at the start of MTX (SD)	58 (14)	52 (15)	57 (15)	54 (15)	61 (14)	52 (14)	63 (13)	54 (15)
Median year of the start of MTX (Q1–Q3)	2011 (07–15)	2011 (05–15)	2010 (06–14)	2010 (05–15)	2015 (11–17)	2015 (13–17)	2016 (14–18)	2015 (12–17)
Median overall number of FDRs (Q1–Q3)	4 (3–5)	4 (3–5)	4 (3–5)	4 (3–5)	4 (3–5)	4 (3–4)	4 (3–5)	4 (3–5)
OR (95% CI)	0.60 (0.23–1.58)		0.71 (0.36–1.41)		0.89 (0.41–1.94)		1.42 (0.66–3.07)	

*DMARD* disease-modifying anti-rheumatic drug, *EULAR* European League Against Rheumatism, *FDR* first-degree relative, *MTX* methotrexate, *OR* odds ratio, *RA* rheumatoid arthritis

^a^Three of these had missing status

^b^One of these had missing status

^c^One of these had missing status

Among the 347 unique individuals, 158 and 119 individuals had an obtainable family history of persistence to MTX in the sense that they all had first-degree relatives starting treatment with MTX in DMARD monotherapy at least 1 (or 3) years prior to their own treatment start. Among the 158 individuals with obtainable family history at 1 year, 108 (68%) were themselves persistent at 1 year and among the 119 individuals with obtainable family history at 3 years, 56 (47%) were themselves persistent at 3 years. Again, cohort characteristics were similarly distributed across groups, except for a later median year of treatment start, as expected per conditioning on the obtainable family history (Table 2). Having a family history of persistence to MTX in DMARD monotherapy was not associated with persistence within the index patient. No association was found at 1 year (OR=0.89, 95% CI 0.41–1.94) nor at 3 years, though the point estimate at 3 years (OR=1.42, 95% CI 0.66–3.07) was comparable to that of the main analysis (RR=1.41, 95% CI 1.14–1.74). Full results from the exploratory analysis, including cohort characteristics, can be found in Table 2.

### Sensitivity analysis

When extending exclusion criteria to include validation of RA diagnosis and treatment persistence against NPR and PDR, 152 patients were excluded from the main cohort. Of these, 114 patients had their first visit listing RA more than 12 months prior to inclusion into SRQ per NPR with 24 patients having filled a prescription (for any condition) of a non-MTX DMARD per PDR. In general, the results from both sensitivity analyses were comparable to the main results.

For familial aggregation of treatment persistence, the point estimates were similar to those observed in the main analysis although the RR for persistence at 3 years was not significant for the cohort that had validation against NPR and PDR (Table 3). For the analysis of heritability, the cohort of full siblings provided higher point estimates compared to the main analysis. Nevertheless, similar to the main analysis, a higher point estimate for the heritability could still be discerned for persistence at 3 years compared to persistence at 1 year (Table 3).

**Table 3:** Sensitivity analysis results. RRs quantifying the familial aggregation of persistence and heritability of persistence based on sensitivity analysis sub-cohorts taken from the main cohort of Swedish early RA patients diagnosed 1999–2019, treated with MTX in monotherapy as their first prescribed DMARD and with a first-degree relative concordant for early RA and treatment with MTX in DMARD monotherapy; the first being a sub-cohort of only full siblings and the second being a sub-cohort within individuals included into SRQ during 2006-2018, where both early RA and first-line treatment with MTX in DMARD monotherapy were validated against NPR and PDR

	Full siblings				Validated against NPR and PDR			
	MTX persistence at 1 year		MTX persistence at 3 years		MTX persistence at 1 year		MTX persistence at 3 years	
	Persistent *N* = 97	Not persistent *N* = 54	Persistent *N* = 73	Not persistent *N* = 78	Persistent *N* = 142	Not persistent *N* = 58	Persistent *N* = 109	Not persistent *N* = 91
RR (95% CI)	1.12 (0.84–1.50)		1.67 (1.16–2.40)		1.07 (0.88–1.31)		1.26 (0.97–1.63)	
*h*^2^ (95% CI)	0.40 (0^a^–0.90)		1.00^a^ (0.65–1.00^a^)		0.23 (0.00^a^–0.73)		0.45 (0.05–0.84)	

*DMARD* disease-modifying anti-rheumatic drug, *MTX* methotrexate, *NPR* National Patient Register, *PDR* Prescribed Drug Register, *RR* relative risk, *RA* rheumatoid arthritis, *SRQ* Swedish Rheumatology Quality Register

^a^Truncated confidence interval boundary

## Discussion

We here present the first investigation of the familial aggregation and heritability of persistence to MTX in DMARD monotherapy in early RA. We did not observe any statistically significant familial aggregation nor heritability during the first year of treatment. However, at 3 years, both estimates were significant, indicating that a familial component, including a possible genetic element, might be present.

We chose to perform a family-based study as this takes the full genetic component of individuals into account (as an extension to the genetic component captured by the markers included in a genome-wide association study). Using persistence as a treatment outcome allows us to circumvent the problem of dependence on the availability of clinical visits with disease activity measures, while still enabling analyses of both short- and long-term outcomes. Previous studies have mostly focused on primary treatment response after 3 or 6 months. Here, we assessed persistence at 1 and 3 years; at 1 year, we capture also those with a slow(-er) response to the drug (compared to assessment at 3 months) and after 3 years we should retain only a minimal number of false-positive responders.

Previous studies have implicated a broad set of genetic variants associated with MTX response [8, 9], as well as adverse events [36], though most variants found through candidate gene approaches have been only weakly associated, or call for replication [37]. Studies have also indicated that models including genetic data predict MTX treatment response better than those relying solely on clinical factors [12, 13]. While the presence of associated genetic variants is an indicator of an existing genetic component, their individual influence on the phenotype is often minor for complex traits. This is commonly taken as a sign of a polygenic trait, meaning that an analysis of genetic variants on an aggregated level, such as through the heritability, may be more informative of the magnitude of the genetic component underlying the phenotype.

When estimating heritability with tetrachoric correlations, an assumption is made that the only similarity between first-degree relatives is due to genetics, thus assuming that potential environmental influence is minor and negligible. Although this assumption is (most likely) false, the magnitude of the individual contributions of genetic and environmental components require further modeling, something the sample size in our study did not allow. Nevertheless, heritability estimates observed with tetrachoric correlations can be regarded as an upper bound of the additive genetic contribution. Setting the precision of the estimates aside, in our sensitivity cohort including only siblings, we observed point estimates that in general were larger than their counterparts for all first-degree relatives. As siblings share an environment during their upbringing, this indicates that not only genetics, but also a shared environmental familial component could have an impact on persistence to treatment.

The present study extends our previous findings in which we investigated familial aggregation of EULAR response to treatment with MTX in monotherapy and tumor necrosis factor inhibitors (TNFi) [11]. Due to small sample sizes, that study could not investigate familial aggregation within MTX-treated individuals, but found a significant familial component related to TNFi discontinuation after 12 months. In general, in RA, larger heritability estimates have been observed for treatment response with TNFi than what is observed in this study for MTX [38–40], something that could be indicative of different treatment response phenotypes for different DMARDs. Studies have also found that different RA treatment outcomes vary highly in heritability estimates and that they in general correlate poorly within outcomes assessed at a single time-point [41], making singular point estimates difficult to compare across treatment type, period and outcome.

Our study has certain limitations. First, as persistence status was assessed based on drugs from the prescribed drugs register, some individuals might be incorrectly classified as they may have chosen to not follow the treatment protocol prescribed by their rheumatologist. However, due to the regular follow-up visits for early RA patients, such discontinuation should generally be captured by the treating rheumatologist leading to only a small number of such patients and a negligible bias. Secondly, as our tetrachoric correlation estimates of the heritabilities could not account for patients with multiple first-degree relatives, the standard errors will be biased leading towards more narrow confidence intervals than expected, had dependencies between observations been accounted for. Despite this, the number of individuals with multiple first-degree relatives was low and their influence on the estimates should be minor. Third, the sample size was a limitation to our study which lead to reduced precision within our estimates. This primarily affected the possibility of analysis of sub-cohorts, leading to sample sizes that were unlikely to produce meaningful inference, such as for analysis of the reason for MTX non-persistence. It should, however, be noted, as previously mentioned, that the cohort used in the study still contains virtually all individuals in Sweden fulfilling the study inclusion criteria. Obtaining a larger sample size using the same unbiased cohort selection is therefore difficult.

Our study has multiple strengths. Our outcome measure of persistence had very low missingness, compared with many outcome studies using composite disease activity measures such as DAS28. This was evidenced in our study by the major decrease in sample size between the primary analysis of persistence and the exploratory analysis of treatment response in the same cohort. Furthermore, the extensive follow-up enabled by the persistence outcome allows for additional capturing of patients with a slower response to MTX, who might have been missed when considering a 6-month follow-up. An analysis that requires complete case data might also suffer from potential inclusion bias, as patients may be assessed differently based on the severity of their disease, something that is avoided through the use of treatment persistence. Moreover, the use of register data from Swedish nationwide population-based registers with near-complete coverage allowed us to estimate valid and precise familial aggregation and heritability as our register-based approach covers virtually every first-degree relative pair in Sweden where both individuals were concordant for early RA and first-line treatment with MTX in DMARD monotherapy.

## Conclusions

In contrast to short-term persistence, long-term persistence to treatment with MTX, here in the form of persistence at 3 years, aggregates within families, and the heritability of this phenotype is rather substantial. These findings imply the existence of a familial component within the long-term persistence to MTX. Whether any such familial component is reflective of characteristics of the underlying RA disease or determinants for MTX response in itself (that is not mediated via primary response) will require further investigation.

## Data Availability

For reasons related to the ethical and legal permits surrounding the study data, these cannot be shared. Requests pertaining to data access can be directed to the senior author.

## Abbreviations

CI: Confidence interval
CRP: C-reactive protein
DAS28: Disease activity score using 28 joint counts
DMARD: Disease-modifying anti-rheumatic drug
ESR: Erythrocyte sedimentation rate
EULAR: European League Against Rheumatism
FDR: First-degree relative
IQR: Interquartile range
MGR: Multi-Generation Register
MTX: Methotrexate
NPR: National Patient Register
OR: Odds ratio
PDR: Prescribed Drug Register
RA: Rheumatoid arthritis
RR: Relative risk
SRQ: Swedish Rheumatology Quality Register
TNFi: Tumor Necrosis Factor inhibitor
